# More variable circadian rhythms in epilepsy captured by long‐term heart rate recordings from wearable sensors

**DOI:** 10.1111/epi.18424

**Published:** 2025-04-26

**Authors:** Billy C. Smith, Christopher Thornton, Rachel E. Stirling, Guillermo M. Besné, Sarah J. Gascoigne, Nathan Evans, Peter N. Taylor, Karoline Leiberg, Philippa J. Karoly, Yujiang Wang

**Affiliations:** ^1^ Computational Neurology, Neuroscience and Psychiatry Lab, School of Computing Newcastle University Newcastle Upon Tyne UK; ^2^ School of Computing, Engineering, & Digital Technologies Teesside University Middlesbrough UK; ^3^ Graeme Clark Institute and Department of Biomedical Engineering University of Melbourne Melbourne Victoria Australia; ^4^ Faculty of Medical Sciences Newcastle University Newcastle Upon Tyne UK; ^5^ University College London Queen Square Institute of Neurology Queen Square London UK

**Keywords:** circadian disruption, day‐to‐day, intraindividual, seizure, variability

## Abstract

**Objective:**

The circadian rhythm synchronizes physiological and behavioral patterns with the 24‐h light–dark cycle. Disruption to the circadian rhythm is linked to various health conditions, although optimal methods to describe these disruptions remain unclear. An emerging approach is to examine the intraindividual variability in measurable properties of the circadian rhythm over extended periods. Epileptic seizures are modulated by circadian rhythms, but the relevance of circadian rhythm disruption in epilepsy remains unexplored. Our study investigates intraindividual circadian variability in epilepsy and its relationship with seizures.

**Methods:**

We retrospectively analyzed >70 000 h of wearable smartwatch data (Fitbit) from 143 people with epilepsy (PWE) and 31 healthy controls. Circadian oscillations in heart rate time series were extracted, daily estimates of circadian period, acrophase, and amplitude properties were produced, and estimates of the intraindividual variability of these properties over an entire recording were calculated.

**Results:**

PWE exhibited greater intraindividual variability in period (76 vs. 57 min, *d* = .66, *p* < .001) and acrophase (64 vs. 48 min, *d* = .49, *p* = .004) compared to controls, but not in amplitude (2 beats per minute, *d* = −.15, *p* = .49). Variability in circadian properties showed no correlation with seizure frequency nor any differences between weeks with and without seizures.

**Significance:**

For the first time, we show that heart rate circadian rhythms are more variable in PWE, detectable via consumer wearable devices. However, no association with seizure frequency or occurrence was found, suggesting that this variability might be underpinned by the epilepsy etiology rather than being a seizure‐driven effect.

**Key points**

- Relative to controls, the circadian rhythm of heart rate was more variable for PWE, measured using commercial wearable devices.
- This provides evidence for long‐term and persistent circadian disruption in epilepsy.
- However, increased intraindividual circadian variability was not related to seizure occurrence or frequency.
- The underlying etiology may instead be associated, or factors such as ASMs or comorbidities may play an important role.

## 1 INTRODUCTION

The circadian rhythm aligns our physiology and behavior to the 24‐h environmental light–dark cycle. A stable circadian rhythm is thought to be important for overall health, and disruptions to the circadian rhythm have been associated with various conditions, including sleep disorders,^1^, ^2^ psychiatric disorders,^3^, ^4^, ^5^, ^6^ and neurological disorders.^7^ However, it remains unclear how circadian disruption should be assessed over the long term; many methods are available depending upon context.^8^, ^9^, ^10^

One promising and easy‐to‐interpret approach is to assess the intraindividual variability of a set of fundamental descriptive properties of the circadian rhythm—for example, amplitude—measured across multiple consecutive days. This approach has also been referred to as "day‐to‐day variability"^11^, ^12^ or "circadian variability."^4^ Intraindividual variability is designed to detect persistent irregularities in circadian rhythms over long‐term physiological recordings. Such data are most easily obtained from peripheral measures that exhibit circadian oscillations using wearable devices (such as heart rate, physical activity, and skin temperature), making this approach a cost‐effective and scalable methodology to study circadian disruption, complementary with existing methods.

Epilepsy is a neurological disorder characterized by recurrent seizures,^13^ which are closely associated with the circadian rhythm.^14^, ^15^, ^16^, ^17^, ^18^, ^19^, ^20^ Sleep patterns are inherently tied to the circadian rhythm, and the relationship between sleep and seizures has been reported upon widely.^21^, ^22^, ^23^, ^24^, ^25^ However, the role of persistent, long‐term circadian disruption in epilepsy remains unstudied. Consequently, we propose the assessment of intraindividual circadian variability in wearable heart rate recordings sourced from people with epilepsy (PWE), which could provide valuable insights for clinical applications, such as enhancing wearable seizure prediction^26^ and guiding chronotherapeutic treatments,^15^, ^20^ including antiseizure medication (ASM) alert systems. Additionally, this novel application of intraindividual variability as a measure of circadian disruption in epilepsy could support its broader use as a wearable clinical biomarker.

Here, we measure the intraindividual variability of three descriptive properties of the circadian rhythm of heart rate using long‐term wearable‐derived heart rate recordings from 143 PWE and 31 controls. We seek to establish whether circadian variability is greater in epilepsy and, if so, whether a relationship exists between variability and seizure frequency and occurrence.

## 2 MATERIALS AND METHODS

### 2.1 Participant data

Wearable smartwatch data from PWE and controls were used retrospectively, sourced from the observational “Tracking Seizure Cycles” study.^14^ Adults with epilepsy were recruited to this study by referral from collaborating epilepsy specialists in tertiary referral epilepsy clinics. Inclusion criteria were diagnosis of epilepsy from a specialized epileptologist, uncontrolled/partially controlled seizures as determined by their neurologist, and that they were deemed capable of keeping a reliable seizure diary. Additionally,^14^ control participants without epilepsy were recruited from their colleagues, friends, and relatives, with no randomization or blinding performed. The study was approved by the St. Vincent's Hospital Human Research Ethics Committee (HREC 009.19). All participants provided written informed consent.

For both PWE and controls, data were collected via a wearable smartwatch (Fitbit), continuously measuring heart rate via photoplethysmography (PPG) at 5 second resolution. Participating PWE also used a mobile device to manually report clinically apparent seizures using the freely available Seer App for either the entirety or a subset of the study period.

Our retrospective analysis of these data was approved by the Newcastle University Ethics Committee (40679/2023).

### 2.2 Measuring circadian disruption using physiological time series: Intraindividual variability approach

**FIGURE 1:**
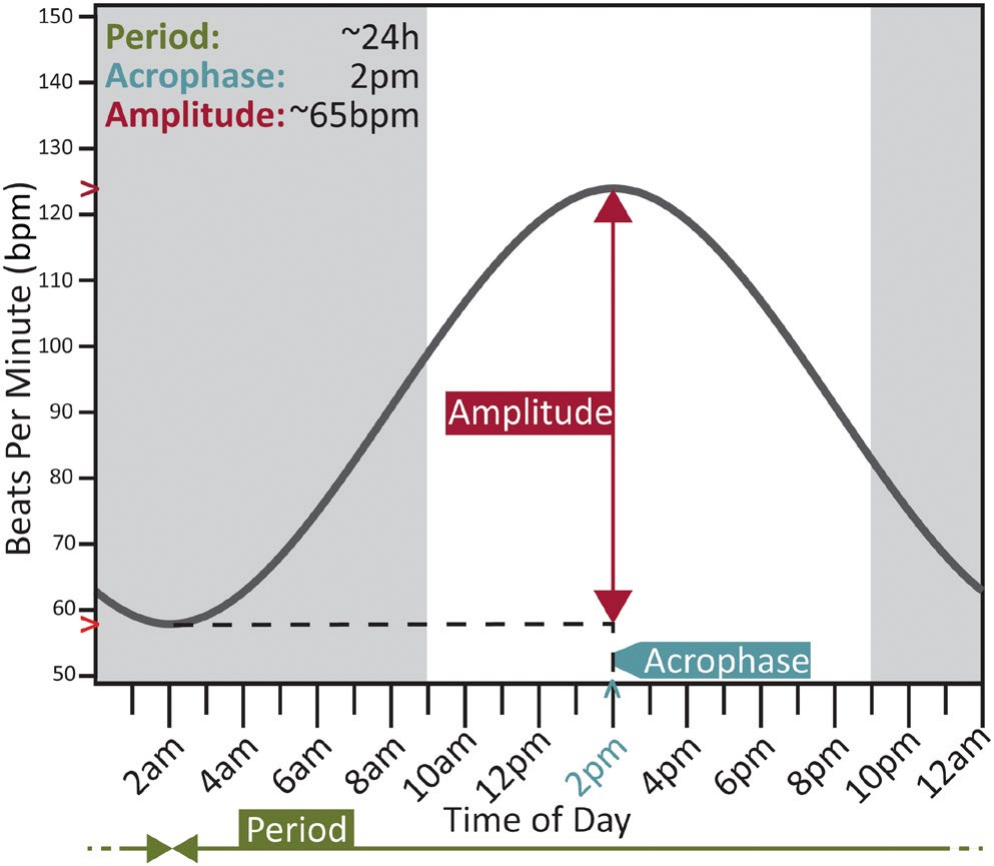
An illustrative schematic of one "cycle" of the circadian rhythm of heart rate (CRHR) with the three descriptive properties used in this study labeled. Period reflects the duration of one cycle of the modeled CRHR (approximately 24 h). Acrophase is defined as the time of day at peak cycle magnitude, reflecting rhythm "timing." Amplitude is a measure of circadian "strength," defined as the difference in magnitude between cycle peak and previous trough. bpm, beats per minute.

**FIGURE 2:**
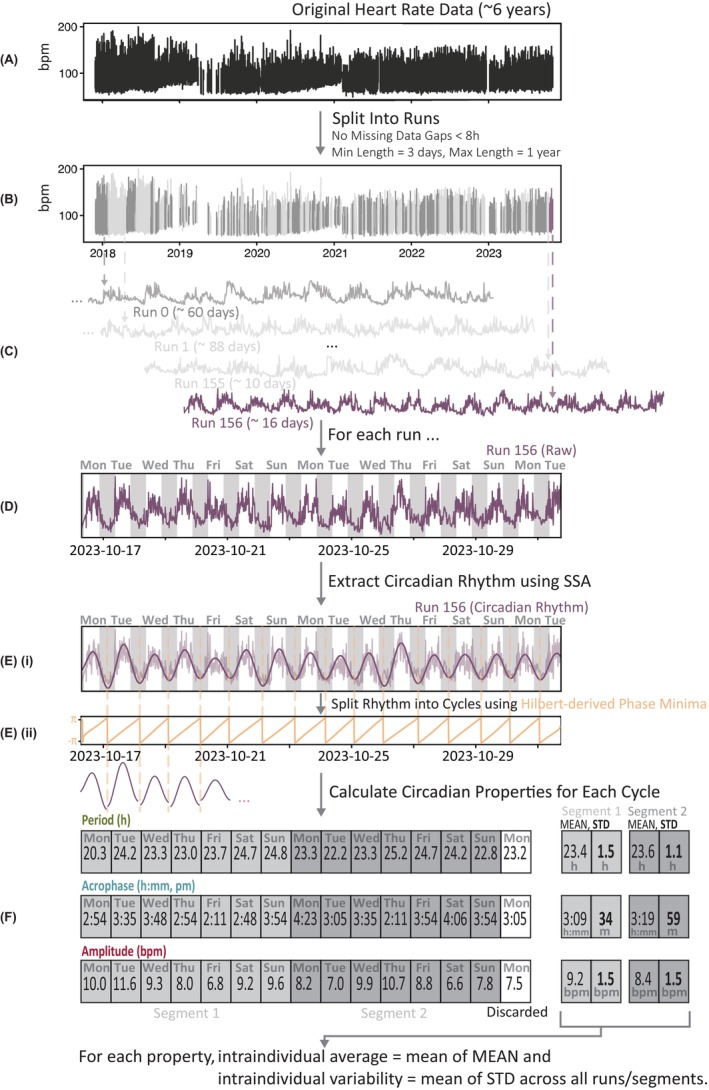
(A, B) Participant heart rate data (A) was split into runs between >8‐h gaps of missing data (B). (C–Ei) Singular spectrum analysis (SSA) extracted the circadian rhythm of heart rate (CRHR) from each run. (Eii) Troughs in the circadian phase were used as reference points for splitting the extracted CRHR into individual cycles. (F) Circadian properties were calculated for each cycle and grouped into segments of 7 days. The mean and SD of each property was then calculated within each 7‐day segment. The mean across all segments in a subject produces the final intraindividual average and variability values. For example, if this sample participant had only these two segments, their intraindividual variability of acrophase would be (34 + 59)/2 = 46.5 min. bpm, beats per minute.

Capturing the intraindividual variability of the circadian rhythm involves the daily measurement of three descriptive circadian properties, which are illustrated in Figure 1. For each participant, the circadian rhythm of heart rate (CRHR) was extracted from their wearable recording. Three circadian properties were then computed for each daily cycle in the CRHR. Finally, estimates of the average (statistical mean) and variability (statistical SD) of each property over the recording were produced. Figure 2 provides an overview of this process, and further details are provided below and in Data S1.

#### 2.2.1 Accounting for gaps in recording

To calculate the circadian properties of the heart rate, a continuous signal was required. Any gaps shorter than 8 h were linearly interpolated, and the signal was split up into "runs" of data between remaining gaps (Figure 2B). Twenty‐six participants (21 PWE, five controls) without any runs longer than 7 days were excluded from further analysis. A more detailed explanation and justification for this approach is provided in Data S1.3.

#### 2.2.2 Extracting circadian rhythm

For each run of data, singular spectrum analysis (SSA) was applied to decompose the continuous raw heart rate time series into components of distinct frequency (Figure 2C–Ei). SSA was found to perform reliably in comparison to similar algorithms (see Data S1.1). To determine which component represented the CRHR, Fourier analysis was performed on each component to determine its central frequency. The component with period (inverse of frequency) closest to 24 h was selected as the circadian component (Figure 2Ei).

#### 2.2.3 Computing circadian properties

The extracted CRHR was then split up into the individual (approximately daily) circadian cycles (Figure 2E), and the period, acrophase, and amplitude were computed for each cycle (Figure 2F). To achieve this, the Hilbert transform was applied to the extracted CRHR, producing a complex‐valued analytic signal, from which the circadian “phase series” (Data S1.2), a measure of circadian cycle progression, was derived (Figure 2Eii). The rhythm was then split into daily cycles at each trough in the phase series. For each individual cycle, *period* was computed as the duration between the first and last time point of the cycle, *acrophase* as the time of day at zero cycle phase, and *amplitude* as the difference in magnitude between acrophase and trough (Figure 1).

#### 2.2.4 Calculating intraindividual variability of circadian properties

Circadian properties for each cycle were grouped into consecutive, nonoverlapping segments of 7 days. The SD was calculated using all 7 days in each segment and property (Figure 2F), and the summary *intraindividual variability* was calculated from the average of the SDs across all segments. The *intraindividual average* of circadian properties was calculated in the same way from the average of the means across segments. The fixed segment size of 7 days prevents any bias introduced by varying recording duration and run length between participants when calculating the mean and SD (see Data S1.3 for more detail).

### 2.3 Statistical analysis

We calculate *p*‐values for reference only, and all reported values are raw, uncorrected by false discovery rate. A nonparametric two‐sided Wilcoxon rank‐sum test was used to compare the intraindividual average and variability of each circadian property between PWE and controls. A nonparametric test was chosen to account for the imbalance between PWE and control sample sizes, but to further control for this, a random subsampling test was performed over 10 000 iterations, where the whole control sample was compared to a random subsample of 28 PWE using the same statistical method as above. Sampling was without replacement within an iteration, but with replacement across iterations.

Pearson correlation was used to test whether an individual's seizure frequency was correlated with their intraindividual variability calculated over segments occurring between the start and end of their seizure diary. Seizure frequency was defined in terms of the average number of seizures per week of recording data postsegmenting. The distribution of seizure frequency was highly skewed, so a log10 transformation was applied for the correlation analysis.

To test whether having one or more seizures in a 7‐day period was associated with greater intraindividual variability, each 7‐day segment occurring within a participant's seizure diary was classified as either seizure‐containing or seizure‐free according to the seizure diary. For PWE with at least five seizure‐free segments and at least five seizure‐containing segments, intraindividual circadian average and variability values were calculated over seizure‐free and seizure‐containing segments separately. These values were then compared using a two‐sided Wilcoxon signed‐rank paired test.

Effect sizes were reported using Cohen *d* throughout.

## 3 RESULTS

Before processing, data were available for *n* = 164 PWE and *n* = 36 controls. After processing, 143 PWE and 31 controls remained for analysis. We analyzed and compared intraindividual variability of circadian rhythm properties (see Materials and Methods) calculated using wearable heart rate data (Fitbit), between PWE and controls.

Briefly, we isolated the circadian rhythm component from the raw heart rate recordings, and measured a range of properties (period, acrophase, amplitude) for each cycle. Intraindividual variability in circadian properties were then obtained across segments of successive cycles. An increased intraindividual variability in any property can be interpreted as a less “stable” circadian rhythm for the individual.

Table S2.1 lists the demographics and other metadata of this cohort. An average of 163 days (SD = 183) were discarded for each subject, resulting in a median recording duration of 215.8 days (interquartile range [IQR] = 580.9) for PWE and 125.1 days (IQR = 260.5) for controls. Although recording duration was shorter in controls compared to PWE, we found no relationship between (postsegmenting) recording duration and intraindividual variability in any circadian property (Data S3). There were no age (*t* = −.215, *p* = .84) or sex (*χ* ^2^ = 2.777, *p* = .427) differences between PWE and controls, and there was no association between intraindividual variability and age, sex, or epilepsy subtype (see Data S5).

### 3.1 Circadian rhythm of heart rate is more variable for PWE

**FIGURE 3:**
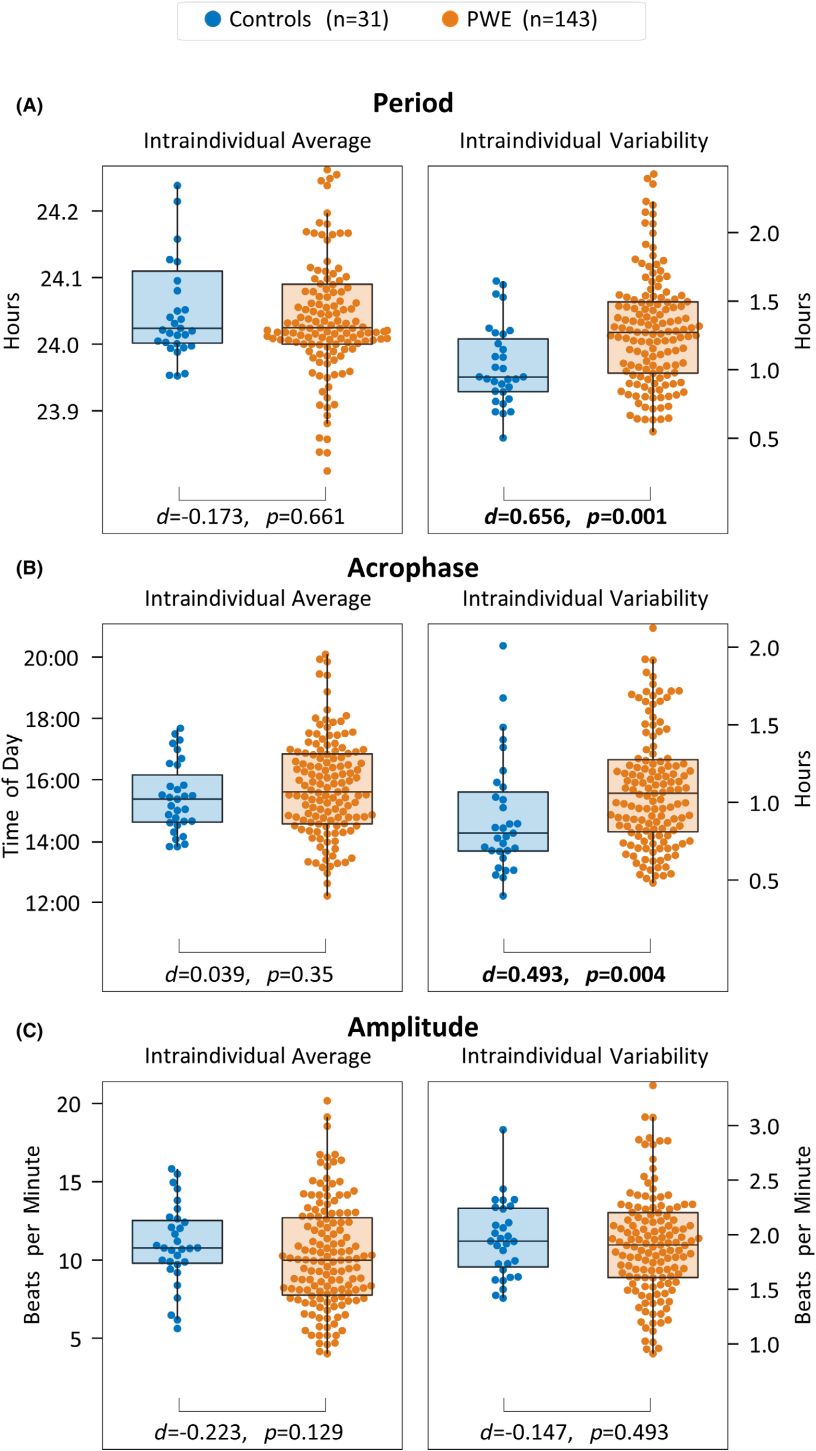
Comparison of the distribution of the intraindividual average and variability of circadian properties between people with epilepsy (PWE) and controls. Period, acrophase, and amplitude are defined in Figure 1. Effect sizes are measured in Cohen *d*, and *p*‐values are based on the two‐sided Wilcoxon rank‐sum test for comparison between PWE and controls. Each subpanel has been zoomed in to omit outliers; outliers were not excluded prior to statistical testing.

The intraindividual variability in period (76 vs. 57 min, *d* = .66, *p* < .001; Figure 3A) and acrophase (64 vs. 48 min, *d* = .49, *p* = .004; Figure 3B) was increased for PWE compared to controls. However, there was no difference in intraindividual variability of amplitude between PWE and controls (∼2 beats per minute, *d* = −.15, *p* = .49; Figure 3C).

For reference, we also present the results for intraindividual average in Figure 3, but we found no substantial difference between PWE and controls (period: *d* = −.17, *p* = .66; acrophase: *d* = .04, *p* = .35; amplitude: *d* = −.22, *p* = .13).

All results held after applying a repeated subsampling test with randomly selected samples of PWE equal in size to controls (see Data S4).

### 3.2 Intraindividual variability does not correlate with increased seizure frequency

**FIGURE 4:**
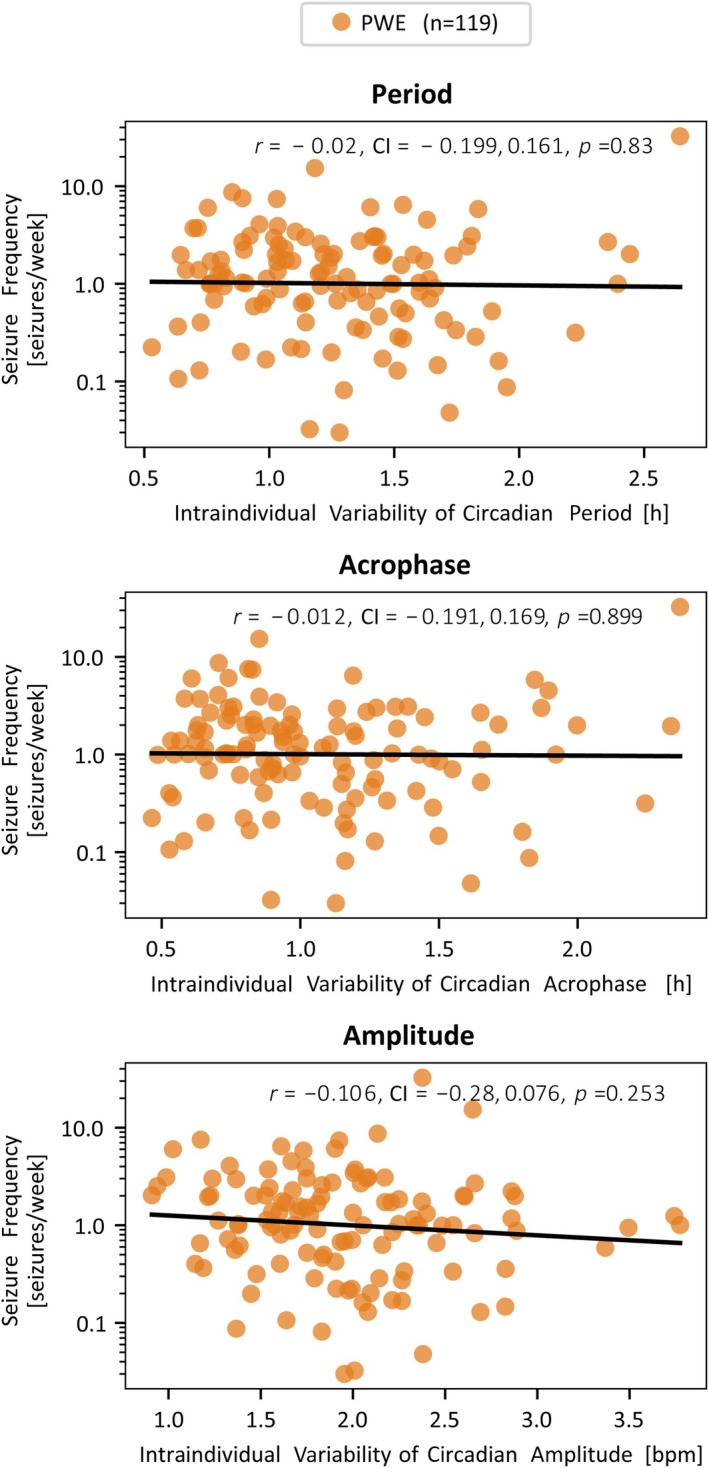
Scatter plots between intraindividual variability of circadian properties and seizure frequency. Seizure frequency is in units of seizures per week on log10 scale. bpm, beats per minute; CI, confidence interval; PWE, people with epilepsy.

Next, the correlation between seizure frequency and intraindividual variability was investigated (Figure 4). PWE without any seizures recorded or with outlying intraindividual variability (absolute *z*‐score > 3) in any property were removed, leaving 119 PWE. We found that intraindividual variability of any property was not correlated with seizure frequency (|*r*| ≤ .1).

### 3.3 Intraindividual variability does not differ between weeks with and without seizures

**FIGURE 5:**
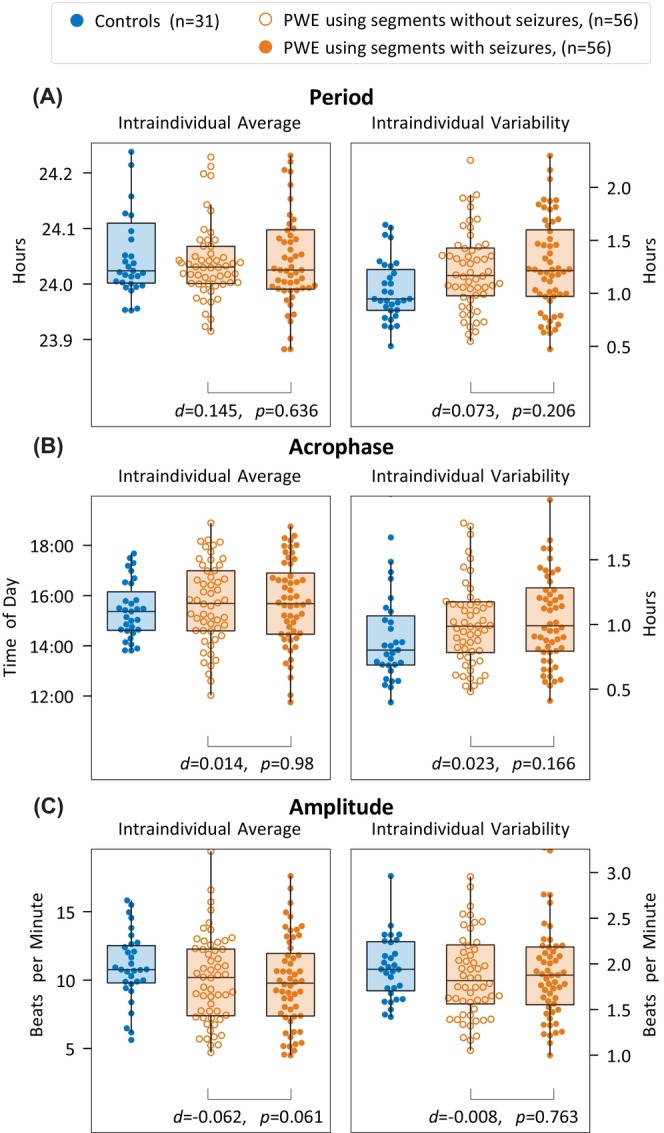
Intraindividual average and variability of circadian properties calculated over segments with and without seizures. Controls are shown for reference and are not included in the statistics shown. The two‐sided Wilcoxon signed‐rank paired test was used for comparison of the intraindividual average and variability calculated using segments with and without seizures. Each subpanel has been zoomed in to omit outliers; outliers were not excluded prior to statistical testing. PWE, people with epilepsy.

We investigated whether intraindividual variability differed between seizure‐containing or seizure‐free 7‐day segments (Figure 5). Individuals without at least five seizure‐free segments and at least five seizure‐containing segments were excluded, leaving 56 PWE. There was no paired difference in intraindividual variability or average of any circadian property between seizure‐free versus seizure‐containing segments.

### 3.4 Weekend and time of year effects

To supplement our findings, we investigated (Data S6) whether weekdays and weekends, as well as the time of year, were associated with the observed increased intraindividual variability in PWE.

We observed a difference between weekends and weekdays in almost all measures (intraindividual averages and variabilities) for both controls and PWE (Figure S6.1). However, the previously observed increases in intraindividual variability in period and acrophase for PWE remained regardless of weekday or weekend (Figure S6.2). This observation was additionally confirmed by a mixed effects model, where we tested for the interaction of weekday/weekend with control/PWE. More details and full statistical models can be found in Data S6.1.

We also tested for a seasonal effect in a similar manner, where we found a less pronounced effect overall. We again confirmed that the observed increase in intraindividual variability in period and acrophase was not driven by seasonal effects or interactions. More details and full statistical models can be found in Data S6.2.

In summary, although weekly and seasonal effects are present in intraindividual averages and variability, these do not drive our main results of increased intraindividual variability in PWE.

## 4 DISCUSSION

This study analyzed the intraindividual variability of three properties of the circadian rhythm using long‐term wearable heart rate data from 143 PWE and 31 controls. We found increased variability in circadian period and acrophase for PWE, providing crucial evidence of circadian disruption in epilepsy. However, we found no evidence of an association between intraindividual variability and seizure frequency, or occurrence, at the population level.

Circadian disruption has a bidirectional relationship with health^27^ and is associated with negative health outcomes^10^ and mortality over the long term.^28^ It is therefore likely to have compounding health implications for PWE, so the results presented here should motivate the development of interventions to reduce the comorbidities associated with circadian disruption for PWE.^29^

We found no evidence of any link between circadian disruption and seizures but consider a link with epilepsy etiology possible. Epilepsy often has a genetic cause,^13^ and many related genes have been identified. Associations between epilepsy and abnormal expression of the genes underlying the circadian rhythm have also been reported.^30^, ^31^ For example, evidence from human ex vivo pathological tissue suggests a link between decreased expression of *BMAL1* and temporal lobe epilepsy^32^—similar findings have been reported for the *CLOCK* gene.^33^ Functionally, the circadian amplitude of neural activity has been found to be reduced in pathological areas using intracranial electroencephalography.^17^ It is possible that any circadian rhythm disruption with a genetic basis at the brain level could be reciprocated at the heart level, either genetically or as a downstream effect of disruption to the central circadian rhythm; such effects may explain the disruption to the circadian rhythm of heart rate for people with epilepsy that we observe here. Moreover, comorbidities of epilepsy such as anxiety and depression^34^, ^35^ are also associated with circadian disruption.^36^ Within the epilepsy cohort, we found that intraindividual variability has a wide range and considerable overlap with the control distribution; future work should investigate the extent to which these comorbidities can explain our findings.

Misalignment of the circadian rhythm with the environmental light/dark cycle, particularly toward the evening, is one form of circadian disruption that has been associated with psychiatric disorders.^6^, ^10^ "Chronotype"—an individual's innate preference as a morning or evening type—relates to such misalignment and has been investigated in epilepsy.^20^, ^30^, ^37^ Precisely reporting on chronotype requires specific methodology so is outside the scope of our study, although average acrophase most closely resembles chronotype. No differences in acrophase were observed between PWE and controls, or between focal and generalized subtypes in our data (Data S5.3). Incidentally, we did observe some differences in circadian rhythms between weekends and weekdays (Figure S6.1) for both PWE and controls. In particular the differences in intraindividual average period and acrophase may be related to the "social jetlag" phenomenon, which refers to differences in sleep and behavioral patterns on weekdays compared to weekends due to the constraints of work, education, or other commitments.^8^ These changes in intraindividual averages were different for PWE compared to controls, which may support previous findings that social jetlag is exacerbated for PWE.^37^ However, most weekday versus weekend effects in intraindividual variability were independent of the control versus PWE effects (Figure S6.2, Table S6.2). Specifically, the highlighted increased intraindividual variability in PWE was not driven by the weekday/weekend effect in our data. It should be noted that insights into social jetlag are limited without inclusion of sleep measures in this study. Future work could test, in a more formal way, the mechanistic relationship between social jetlag, chronotypes, sleep, and our proposed measures of circadian variability.

Interestingly, in this study we observed that the median circadian period was marginally longer than 24 h (24.02 h) for both controls and PWE. In constant laboratory conditions without time cues such as light/dark cycles, the circadian rhythm persists in humans, but with a period of up to 25 h.^38^ In a laboratory setting where light/dark cycles are fixed,^39^ a variable circadian period of temperature and melatonin rhythms between participants, above and below 24 h, was reported that was shorter (24.09 h) on average for females than males (24.19 h). Our study was undertaken under no such controlled conditions; circadian period would certainly be entrained by light and other environmental cues, which may explain why our reported average is closer to 24 h. Additionally, we did not observe any difference in average circadian period between males and females (Data S5). Shifts in average circadian period may be associated with modulatory effects of longer, multiday rhythms of heart rate,^14^ or associated with the annual variations of circadian properties that we also observed (Section S6.2). Future work could explore the dynamics of circadian period over time in detail and whether re‐entrainment differs between PWE and controls.

Our approach has some key limitations. First, although heart rate follows a strong circadian cycle,^40^ bouts of activity such as exercise or stress can cause "behavioral masking," interfering with measurement of the circadian state,^41^ particularly when derived from wearable PPG. Despite the issues of noise, resolution, and accuracy associated with wearable devices, they provide key advantages in affordability, convenience, and scalability over traditional methods for measuring the circadian rhythm such as melatonin sampling, enabling their wider use in clinical contexts and normative benchmarking. Future work should take advantage of multimodal wearables, integrating heart rate with activity and temperature for circadian state estimation, for example. Second, our analysis does not include some key variables that may play a role in the intraindividual variability we observe. ASMs have been reported to both stabilize and disrupt sleep,^22^, ^23^ and so could explain the increased intraindividual variability we see in PWE as well as the overlap with controls if this effect translates to the circadian rhythm more generally. Demographics such as age and sex are also known to associate with measures of the circadian rhythm.^7^, ^42^ In a subset of the cohort (Data S5), we did observe a correlation between age and intraindividual averages of acrophase and amplitude, as expected based on this previous literature, but not with variability in any property. We did not find any sex differences with respect to circadian average or variability. Future work should further clarify whether associations with these potential covariates can be ruled out.

Finally, the role of sleep disruption in epilepsy is well noted,^15^, ^16^, ^23^, ^24^, ^43^, ^44^, ^45^ and although it is a distinct process physiologically,^8^, ^16^, ^46^ it is intrinsically tied to the circadian rhythm. As the circadian behavior of heart rate is tied to sleep, its variability may primarily be driven by sleep disruptions. Future studies should incorporate sleep quality questionnaires and wearable sleep tracking to distinguish between sleep‐related variability versus broader intraindividual variability of the circadian rhythm.

## 5 CONCLUSIONS

In conclusion, we found increased variability of the circadian rhythm of heart rate in epilepsy, which may be indicative of a pathological circadian rhythm disruption in epilepsy, as detected using a commercial wearable device. The effect driving this remains unclear; we were unable to detect any relationship with seizures, so we instead propose that comorbidities, ASMs, sleep disruption, or some other confounds may be involved, or that it is an additional effect—alongside seizures—of the cellular dysfunction underlying the etiology found in some epilepsies. We hope our findings encourage further research of this phenomenon and whether it has application in seizure prediction and chronotherapy in epilepsy, and promote the use of intraindividual variability of circadian properties as a wearable biomarker for disruption in other conditions.

## AUTHOR CONTRIBUTIONS

Billy C. Smith contributed to conceptualization, methodology, software, formal analysis, writing, and visualization. Christopher Thornton contributed to methodology, software, writing, visualization, and supervision. Rachel E. Stirling contributed to resources, data curation, validation, and writing. Guillermo M. Besné and Nathan Evans contributed to validation and supervision. Sarah J. Gascoigne contributed to validation and methodology. Peter N. Taylor contributed to writing, visualization, and supervision. Karoline Leiberg contributed to methodology, validation, writing, and supervision. Philippa J. Karoly contributed to resources, data curation, validation, and writing. Yujiang Wang contributed to conceptualization, methodology, software, formal analysis, writing, visualization, supervision, project administration, and funding acquisition.

## CONFLICT OF INTEREST STATEMENT

None of the authors has any conflict of interest to disclose. We confirm that we have read the Journal's position on issues involved in ethical publication and affirm that this report is consistent with those guidelines.

## Supporting information


DATA S1

## Data Availability

All wearable heart rate recordings and seizure metadata are available upon request to P.J.K. (karoly.p@unimelb.edu.au). A subset of these data is already publicly available on Figshare (DOI 10.26188/15109896) from a previous publication.^14^ Analysis code is available on GitHub: https://github.com/cnnp‐lab/2024_Billy_Intra‐individual_variability_circadian.
