# Supplementary material for: More variable circadian rhythms in epilepsy captured by long‐term heart rate recordings from wearable sensors
Source: Epilepsia. 2025 Apr 26;66(8):2754–65. doi: 10.1111/epi.18424 (PMC12371681; doi:10.1111/epi.18424)
Supplement: Supplementary file 1 — DATA S1 [file EPI-66-2754-s001.pdf]

Supplementary

S1 Methodological details

S1.1 Comparison of circadian rhythm extraction methods

Approaches for computational modelling of the circadian rhythm broadly fall into two categories: dynamic modelling, where the behaviour of the circadian system is represented within a set of equations, and statistical modelling, where a periodic function approximating an underlying core circadian fluctuation is fit to, or derived from, the data (9). Here, we apply the latter statistical modelling methods.

The simplest statistical modelling approach involves the fitting of a sinusoid wave. The rhythm produced is fixed in period, acrophase and amplitude. However, it can often be empirically observed (especially in uncontrolled conditions) that these properties vary over time from one circadian cycle to the next (9), potentially as the result of challenges to the circadian system (e.g inconsistent night shift working), variation in lifestyle and behaviour (e.g occasional late nights), or perhaps even health conditions (e.g the change between mood states in bipolar disorder, or seizure occurrence in epilepsy).

Bandpass-filtering of the signal around 24 hours derives a circadian rhythm where amplitude is able to vary across cycles, but the other parameters remain somewhat fixed. More sophisticated methods exist, such as wavelet-based analysis (the continuous and discrete wavelet transform), signal decomposition techniques (such as empirical mode decomposition) and others such as singular spectrum analysis (SSA) (Figure 2E(i)). An overview these methods can be found in (47). These methods are more flexible and produce a rhythm that fits the data better, capturing variation in all three properties across cycles.

(12) evaluated these as well as other methods for extraction of a circadian rhythm from a set of wearable measures (activity, heart rate, blood pressure, skin temperature, core temperature) recorded over one week from one individual. For each method and measure, they extracted a circadian rhythm and computed the goodness-of-fit (R^2) of the rhythm extracted compared to

the original timeseries. While certain methods performed better on some measures, SSA was the best all-rounder across measures. We perform our own comparison using the same approach in Supplementary Figure S1.1, though with a smaller subset of methods and only using heart rate, but crucially we test over a much larger cohort (31 controls and 143 PWE). SSA performs best across methods (average $R^2 \approx 0.4$). Interestingly, there is a persistently lower R^2 across measures for PWE, potentially supportive of a more variable rhythm for PWE that is picked up poorly by less flexible methods (BANDPASS, COSINE), though could also be related to the increased sample size and recording duration of PWE - notably this difference is minimised for SSA. This finding further informs us that, on average, 40% of the variability in heart rate timeseries was accounted for by the circadian rhythm in this study, the remaining 60% associated with other factors, potentially exercise or acute stress.

While (12) found that certain methods (EEMD, CEEMDAN) performed better in heart rate, the sample size of 1 individual should be noted, as our result shows that, between participants, there is considerable variation and overlap in the R^2 between methods. For this reason, as well as SSA's reliable performance in both (12) study and our own analysis, and our previous experience with these methods, we selected SSA for use in this study. Future work should compare a larger set of methods more thoroughly across a larger and more varied population.

S1.2 Deriving circadian cycles for computing circadian properties

We split the rhythm into the individual circadian cycles (Figure 2E(ii)), and compute the daily period, acrophase and amplitude (Figure 2G). To do so, the Hilbert transform is applied to the extracted circadian rhythm, producing a complex-valued analytic signal, from which the circadian "phase series" can be derived (Figure 2E(ii)). The phase series is a periodic triangular waveform with bounds of $-\pi$ to π. It is aligned with the input circadian rhythm, and can be thought of as a measure of circadian progression; at $-\pi$ the rhythm is at trough, and reaches its peak as phase increases to 0. As phase increases further from 0 to π, the cycle falls again before it reaches the next trough at π, where it wraps back around to $-\pi$. Using this, we can robustly split the rhythm at each phase trough ($-\pi$) into the daily circadian cycles. Once we have collected individual cycles,

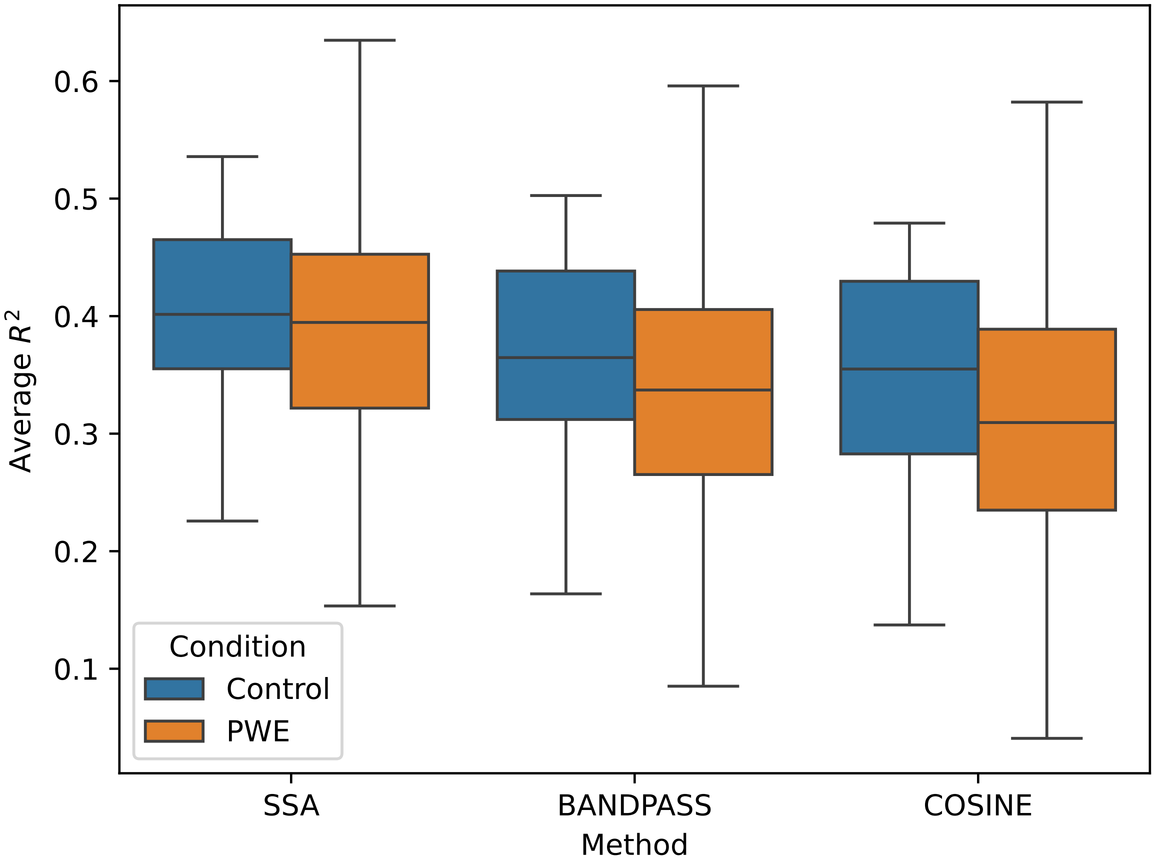

Figure S1.1: The distribution of goodness-of-fit (R^2 (derived from Pearson's r) averaged across runs for each participant) of the circadian rhythm extracted by each method compared to the participant's original heart rate timeseries, split by PWE and controls. For methods requiring a minimum/maximum period range for the circadian rhythm the be detected within (CWT, BANDPASS), 20-30 hours were used respectively. This is the same threshold used to identify the circadian rhythm in the set of components extracted by SSA.

the circadian properties (Figure 1) are calculated for each cycle.

S1.3 Accounting for gaps in recording and variability in recording duration between participants

As shown in Suppl. Table S2.1, the recording duration for both PWE and controls in this cohort is uniquely long, and there is substantial variation in recording duration between participants. These factors necessitated two additional steps in the calculation of intra-individual variability: the splitting of a participant's recording into "runs" between missing gaps prior to extraction of the circadian rhythm, and the grouping of extracted per-cycle circadian property values into "segments" prior to computation of mean and standard deviation.

Missing data gaps in the recording were most likely caused by device charging or removal of the device by the participant for whatever other reason. As the heart rate recordings were computed by a proprietary Fitbit algorithm from raw PPG, it is possible that periods of noisy data (for example, due to poor device contact with skin) were excluded automatically also. SSA cannot handle missing data in the input recording, so interpolation (e.g linear) is required. However, there are occasionally very long (days-months) gaps of missing data for some participants, so we opted to avoid running the algorithm over very long interpolations of data, as this wasted computational resources and interfered with results. As such, the raw heart rate data was split into 'runs' between missing gaps larger than 8 hours. Within each run, any remaining gaps (which as such must be below 8 hours) were linearly interpolated.

As stated previously, the intra-individual variability approach involves computation of a mean and standard deviation for each of the circadian properties (period, acrophase, amplitude) for each participant. However, given the variation in recording duration between participants (and especially between PWE and controls) in this dataset, and given that estimation of mean and standard deviation become more reliable as sample size increases, it would not be fair to compare the intra-individual variability between a participant with, for example, a recording duration of a month

to a participant with multiple years worth of data. Therefore, once the circadian properties have been calculated for each cycle, rather than calculating the mean and standard deviation of each property across *all* cycles, we group cycles into consecutive non-overlapping seven-day 'segments'.

Grouping occurred within runs; for example, a run that contained 17 circadian cycles would produce 2 segments (14 cycles), with 3 cycles discarded. We opted to perform the segmenting within runs rather than across the cycles of all runs as, given the minimum 8 hour gap between runs, there may be a considerable gap of time between two consecutive runs.

For each seven-day segment, the standard deviation of each property (Figure 2G) was calculated, reflecting the variability of that property during the corresponding week. Therefore, for each participant, a distribution of standard deviation values is produced for each property, reflecting differences in variability across weeks. To summarise these distributions for each participant, three summary values - intra-individual circadian period variability, acrophase variability and amplitude variability - were calculated by taking the mean of the standard deviation values of each property across the segments distribution. Three additional summary values: intra-individual period average, acrophase average and amplitude average, were calculated in a similar manner, using the mean of each property across all segments.

Calculating the mean and standard deviation separately for each segment ensures that they are always calculated on samples of data of the same length (7 cycles), reducing the problem of varying recording duration between participants. Taking the mean across all segments allows for comparison of circadian average and variability between participants.

A visualisation of our implementation of the intra-individual variability method, with the addition of the segmenting step, can be found in Figure 2.

S2 Demographic and clinical information table

	Controls	Epilepsy	Statistic	p-value
**N**	31	143		
**Age** mean (years)	37.0	38.6	-0.215	0.84
**Age** sd (years)	16.9	13.3		
**Age** unavailable	26	39		
**Sex** female	15	87	2.777	0.427
**Sex** male	13	39		
**Sex** other	0	1		
**Sex** unavailable	3	16		
**Recording Duration** mean (days)	221.5	443.9	1686	**0.037**
**Recording Duration** sd (days)	256.4	531.1		
**Recording Duration** median (days)	125.1	215.8		
**Recording Duration** IQR (days)	260.5	580.9		
**Recording Duration** (min, max) (days)	(7.0, 946.0)	(6.7, 2715.6)		
**Focal**		41		
**Generalised**		13		
**Mixed**		3		
**Total Number of Seizures**		6110		
**Average Number of Seizures**		43.4		

Table S2.1: Demographic and clinical characteristics of the cohort. The distribution of age was compared between PWE and controls using the independent two-sided Welch's t-Test. Sex comparison was performed using the Chi-square test of independence. Duration comparison was performed using the two-sided Mann-Whitney U test.

S3 Recording duration does not affect intra-individual variability estimates

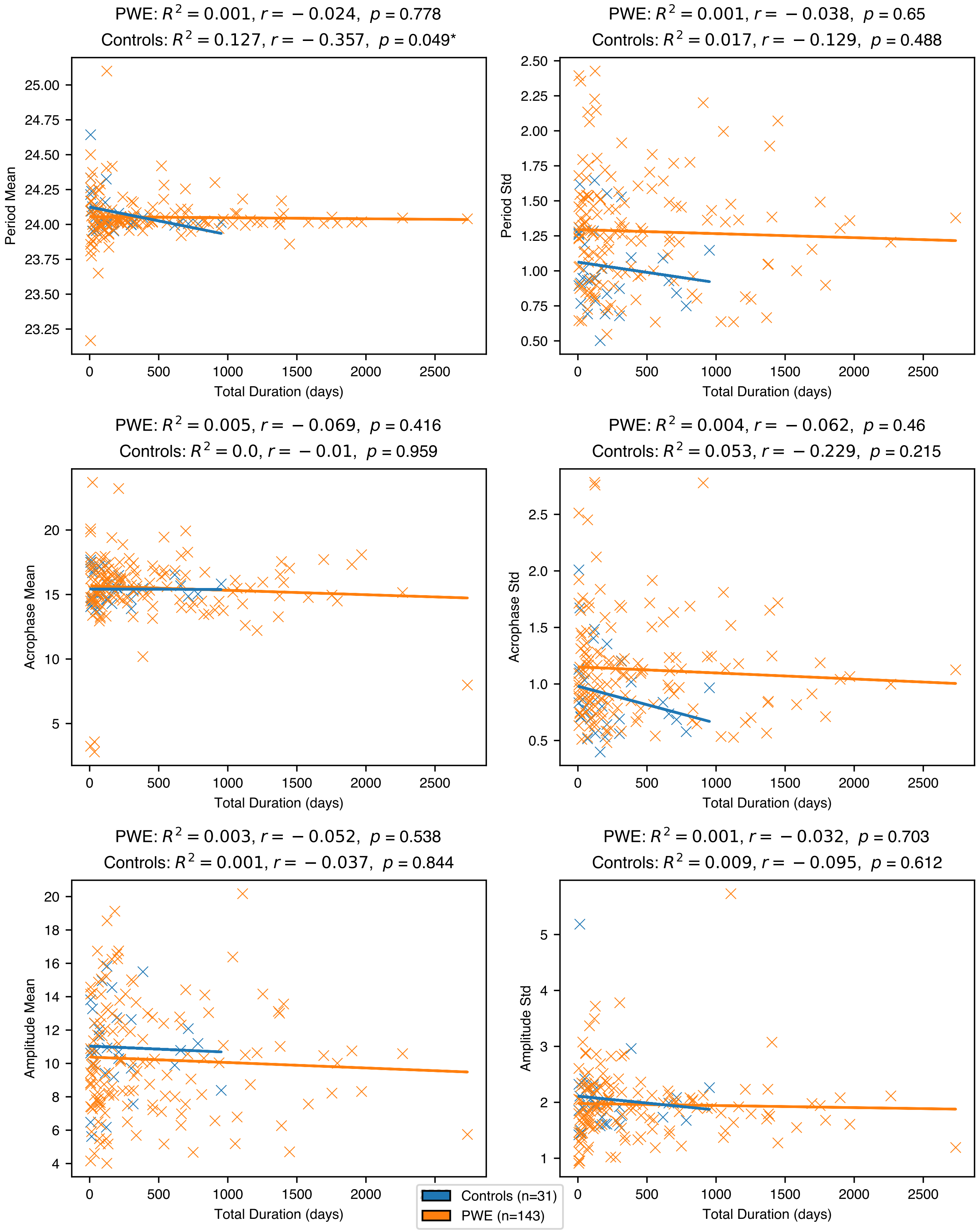

Figure S3.1: The relationship between intra-individual variability of circadian properties and total duration.

S4 Results hold when we resample to account for unbalanced samples

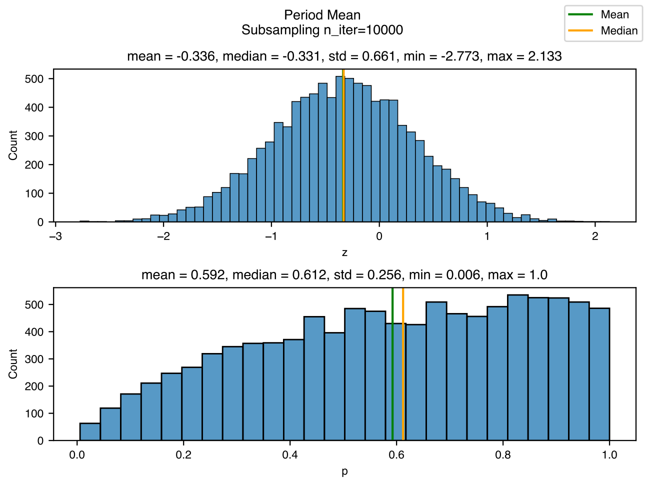

(a) Period Mean

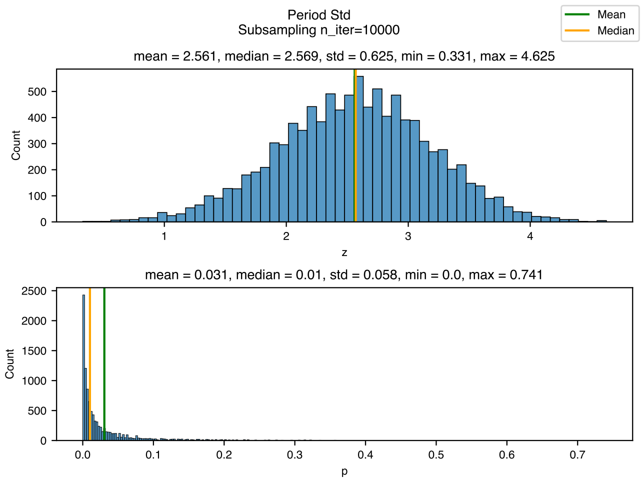

(b) Period Std

	median z	median p
Period Mean	-0.331	0.612
Period Std	2.569	**0.01**
Acrophase Mean	0.739	0.435
Acrophase Std	2.231	**0.026**
Amplitude Mean	-1.19	0.234
Amplitude Std	-0.528	0.531

(c) Table of random sub-sampling median p-values and z-statistics

Figure S4.1: Overview of the random sub-sampling correction to Figure 3. (a) and (b) show the distribution of the z-statistic and p-value over 10,000 iterations of the Wilcoxon rank-sum test using the entire Control cohort and a randomly selected sub-sample of 31 PWE. (a) shows this distribution for Period Mean (intra-individual average of period), which was not originally different between PWE and controls. This result holds after applying random sub-sampling as the z-statistic is normally distributed around ∼0 (no effect) and the p-value distribution is uniform. (b) shows this distribution for Period Std (intra-individual variability of period), which was originally different between PWE and controls. This result also holds after applying random sub-sampling, as the z-statistic is normally distributed around 2.5 (moderate effect) and the p-value distribution is extremely skewed with a median < 0.05. (c) shows the median z-statistic and p-values across each property average and variability.

S5 Increased intra-individual variability of circadian properties is not age, sex or epilepsy type dependent

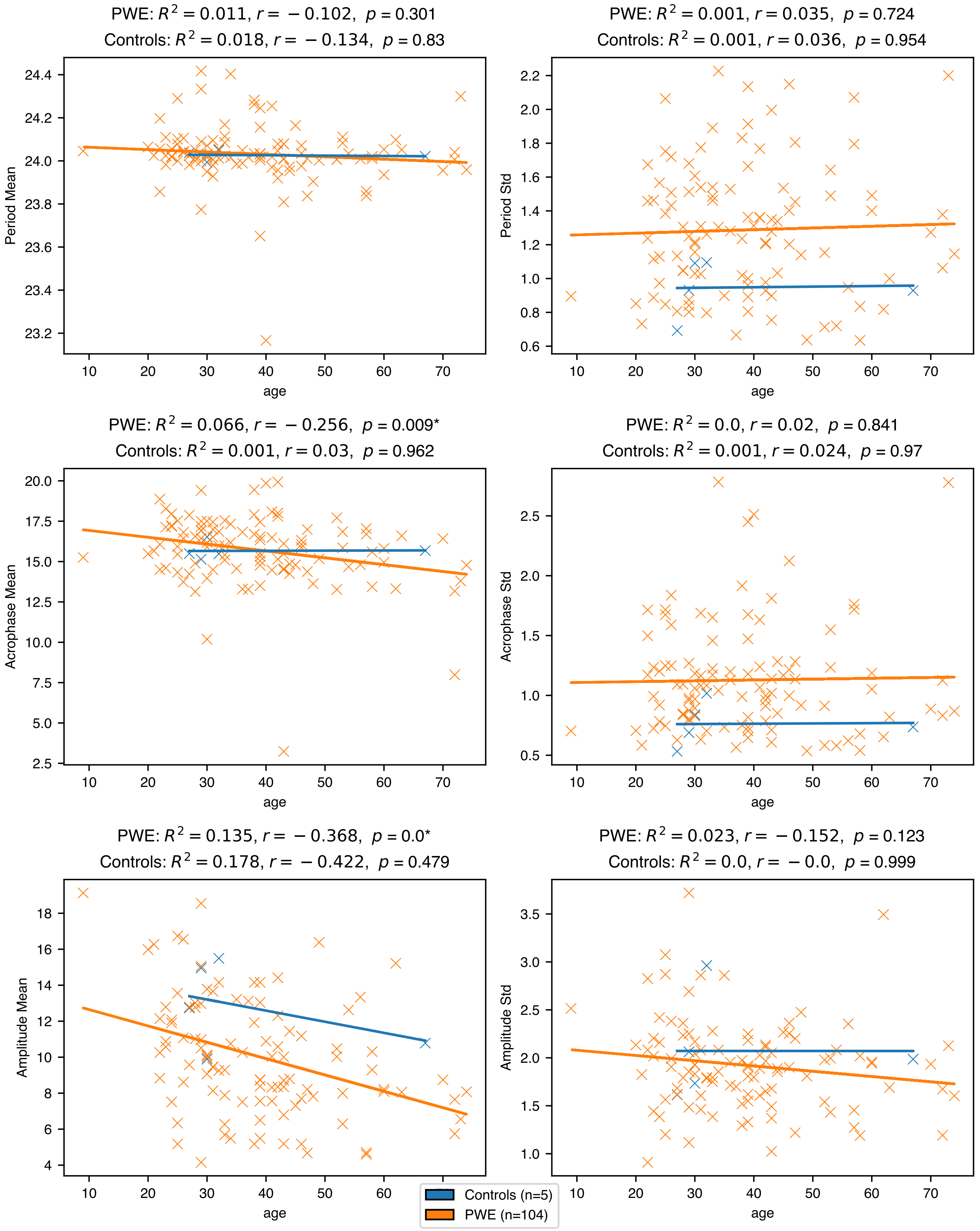

Figure S5.1: The relationship between intra-individual variability of circadian properties and averages and age.

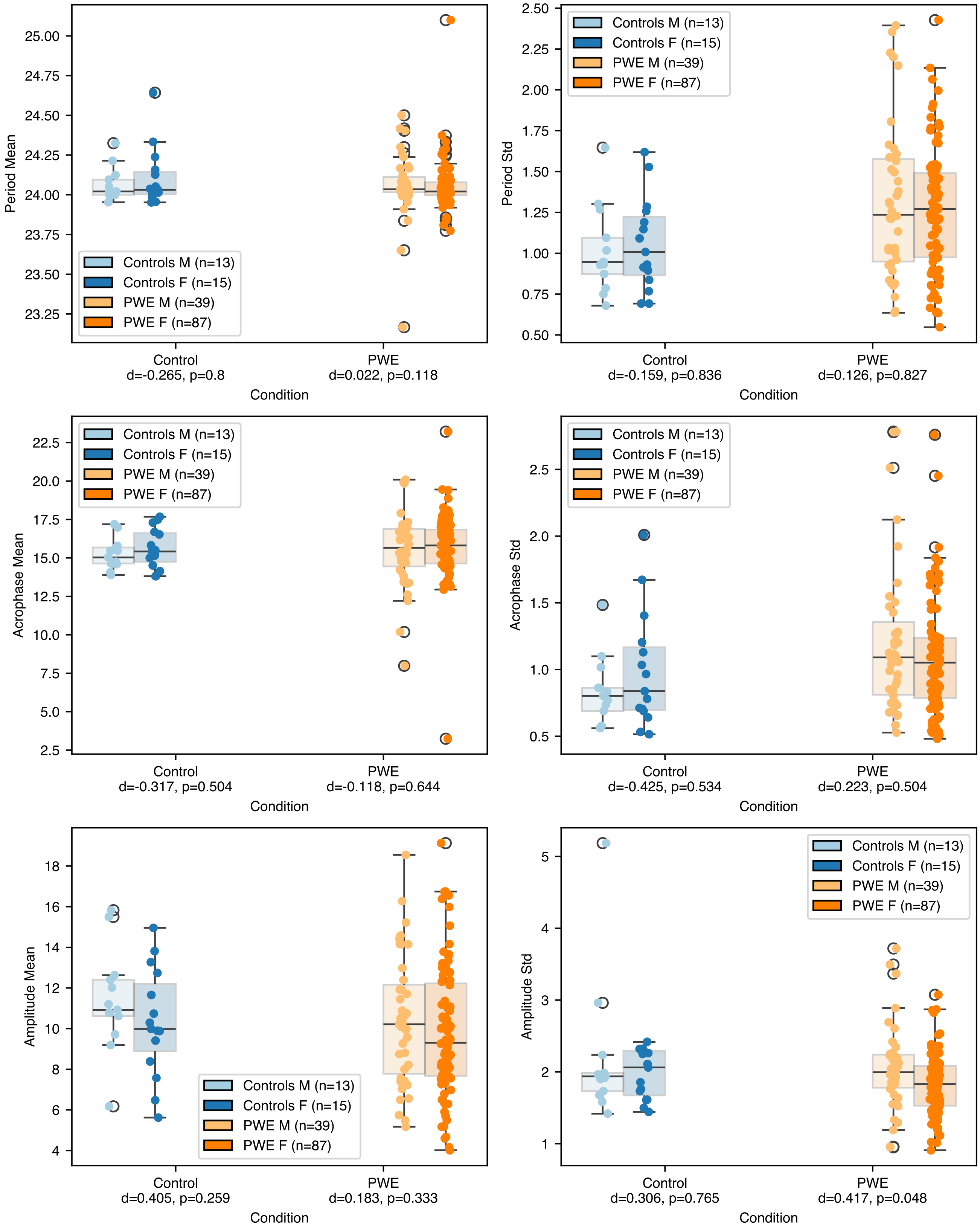

Figure S5.2: Comparison of the distribution of the average and variability in each circadian property between PWE and controls, split by sex. Two-sided Wilcoxon rank-sum test used to compare circadian average and variability between males and females within either PWE or controls.

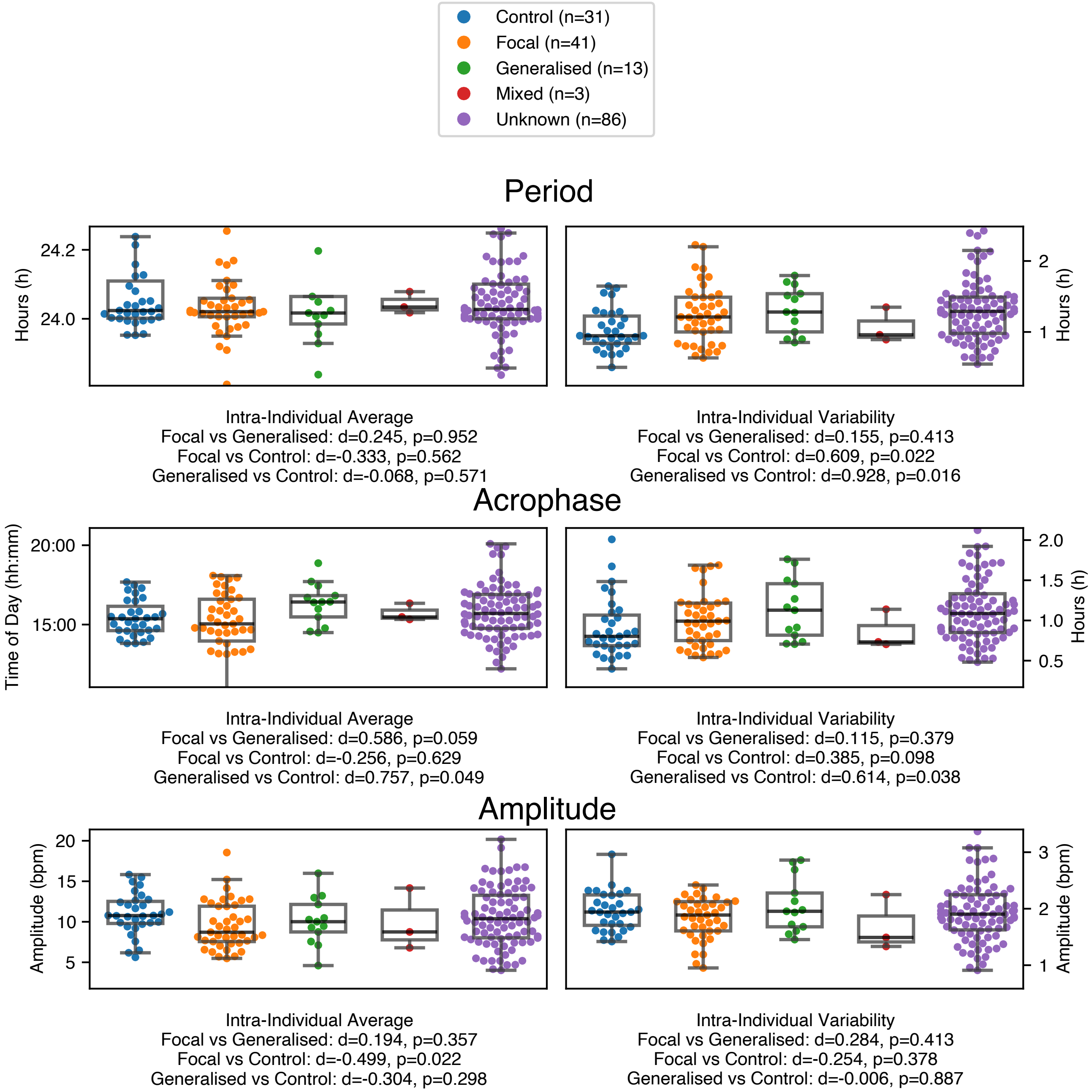

Figure S5.3: Comparison of the distribution of the intra-individual average and variability of circadian properties between epilepsy types. Mixed type and unknown (not provided) type PWE are shown for reference only and are not included in the statistics shown. The two-sided Wilcoxon rank-sum test was used for comparison of the intra-individual average and variability between generalised and focal epilepsy, and each sub-type against controls. Each subpanel has been zoomed-in to omit outliers; outliers were not excluded prior to statistical testing.

S6 Impact of weekends and time of year on circadian properties and their intra-individual average and variability

To investigate whether variability in the circadian rhythm is associated with weekday *vs.* weekend effects or seasonal variations, circadian cycles (Figure 2G) were associated with a date (at acrophase), and 7-day segments were associated with their 'time of year' (see below).

S6.1 Weekday vs weekend effects

'Social jetlag' refers to the phenomenon where sleep patterns differ on weekdays compared to weekends due to the constraints of work, education or other commitments (8). More pronounced social jetlag has been reported for PWE compared to controls (37). Sleep patterns, while distinct from the circadian rhythm, are linked. As such, we test here whether circadian variability is similarly associated with weekday/weekend differences, and whether this varies between PWE and controls.

To compare intra-individual circadian average and variability between weekends and weekdays, each segment was split into 'mid-week' (Tue, Wed, Thu) and 'circa-weekend' (Fri, Sat, Sun) subsegments, and intra-individual circadian average and variability values were calculated over mid-week and circa-weekend segments separately. The equal 3-day segment size was selected as a fully weekday/weekend (Mon-Fri vs Sat-Sun) comparison introduces a sample size bias (5 vs 2) that interferes with mean and standard deviation interpretation. Furthermore, we hypothesize this may account for behavioural shifts - individuals who experience social jetlag are most likely adjusted to their weekday schedule by Tuesday (following a disruptive Monday), and there is maybe a shift in behaviour on Friday evening compared to the evenings on days prior. Firstly, intra-individual circadian average and variability is compared between weekends and weekdays within PWE and control groups separately in Figure S6.1 to determine if a 'weekend effect' exists for both groups or is unique to either. Following this, Figure S6.2 compares intra-individual circadian average and variability between PWE and controls on weekdays and weekends separately, to determine if previously observed differences in these properties between PWE controls are consistent across the

week, or are being driven by a weekend effect.

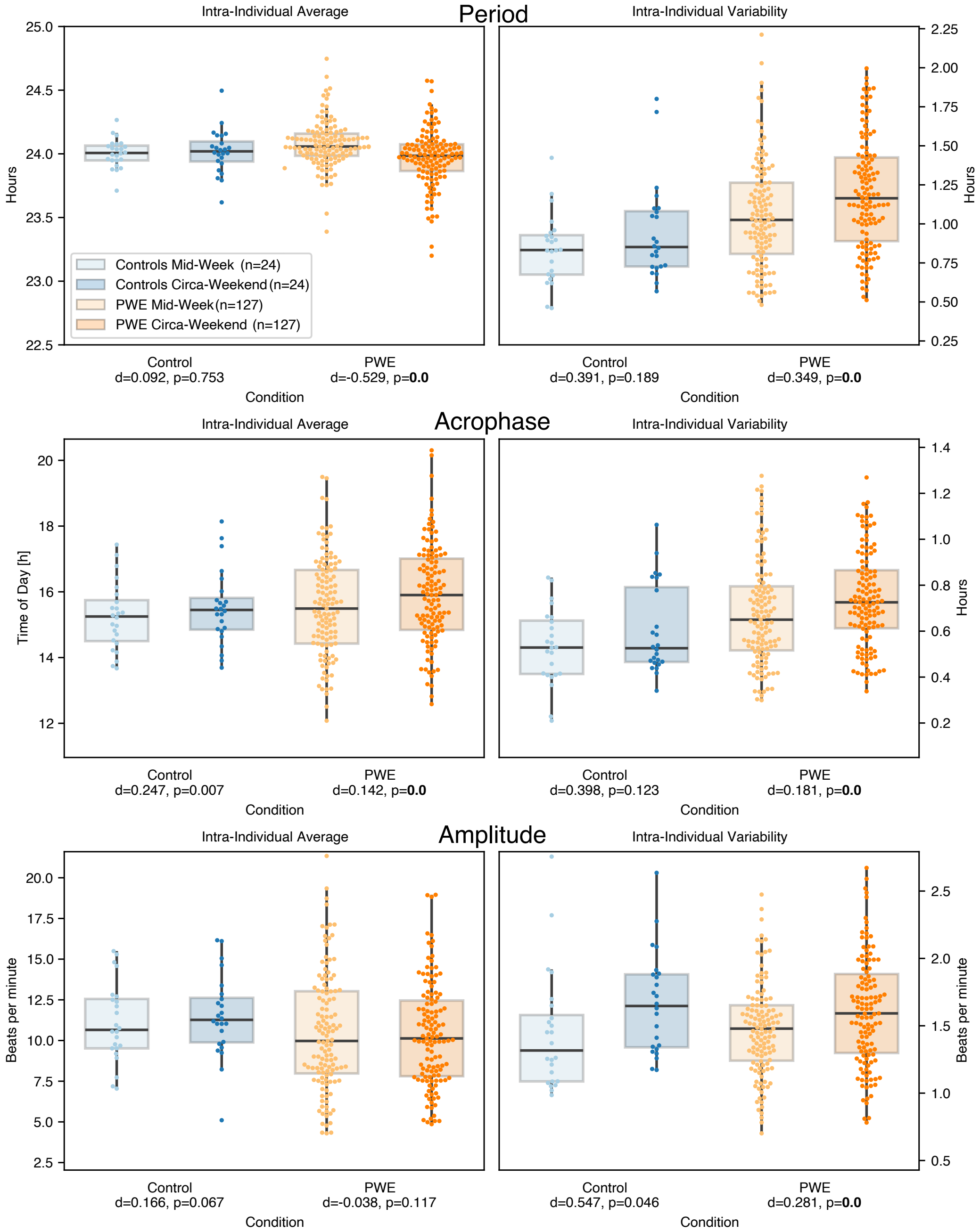

Figure S6.1: Intra-individual average and variability of circadian properties calculated over mid-week (Tue, Wed, Thu, lighter colours) and circa-weekend (Fri, Sat, Sun, darker colours) segments separately for both PWE and controls. The two-sided Wilcoxon signed-rank paired test was performed for comparison of the intra-individual average and variability calculated using mid-week vs circa-weekend segments for PWE and controls independently (i.e, **is there a weekend effect within each group?**). Only participants with at least 3 mid-week and at least 3 circa-weekend segments were kept. Highlighted p-values fall below the Bonferroni-corrected alpha threshold of $0.05/12 = 0.0042$.

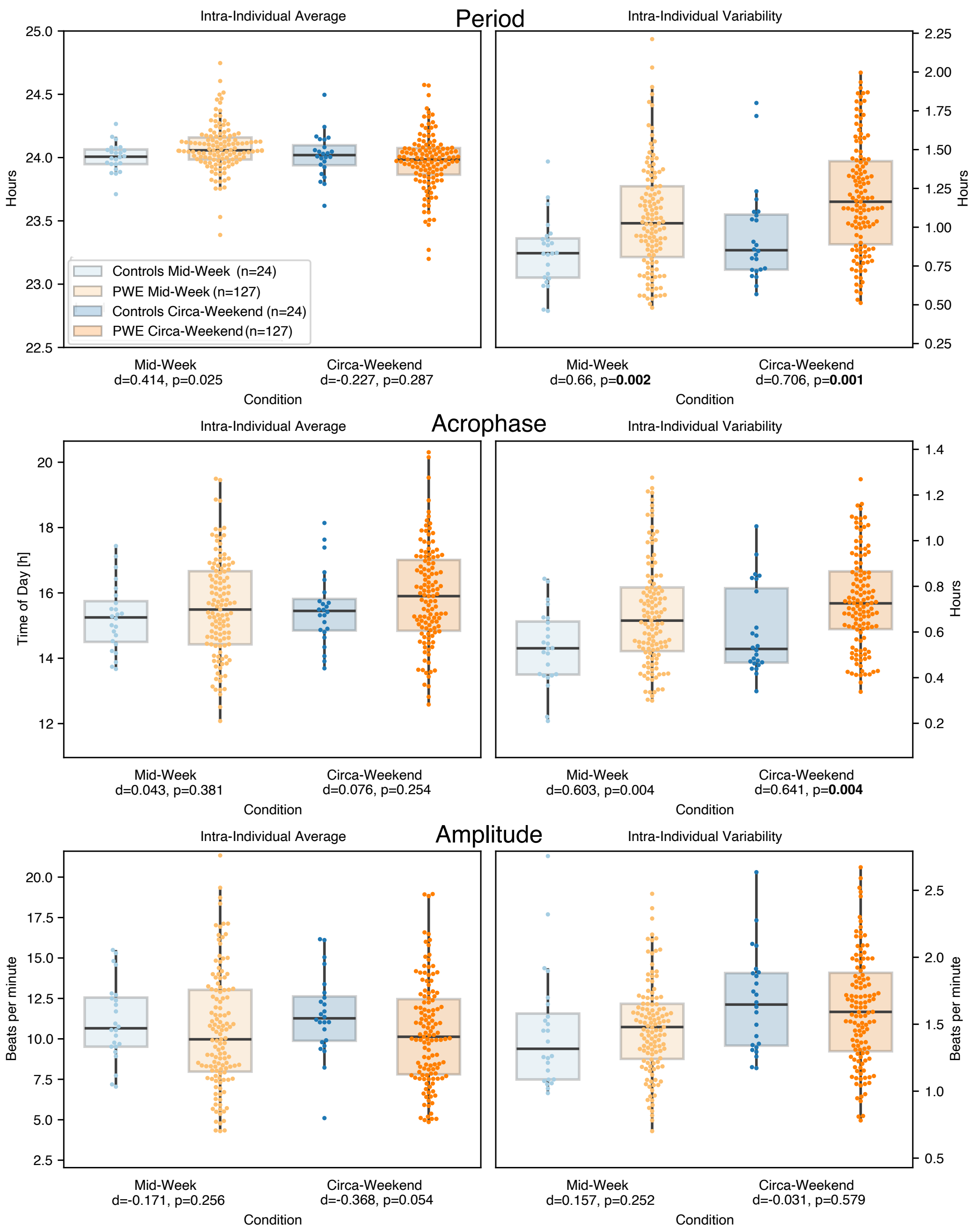

Figure S6.2: Intra-individual average and variability of circadian properties calculated over mid-week (Tue, Wed, Thu, lighter colours) and circa-weekend (Fri, Sat, Sun, darker colours) segments separately for both PWE and controls. The two-sided Wilcoxon rank-sum test was performed for comparison of the intra-individual average and variability calculated using mid-week vs circa-weekend segments separately, between controls and PWE (i.e, **is there a weekend effect between groups?**). Only participants with at least 3 mid-week and at least 3 circa-weekend segments were kept. Highlighted p-values fall below the Bonferroni-corrected alpha threshold of $0.05/12 = 0.0042$.

To further explore the association between intra-individual average and variability of circadian properties in epilepsy and the weekend effect and determine the relative importance of these factors, a mixed-effects linear regression (MELR) model was used:

$$\begin{aligned} property\_measure \sim & total\_duration\_d \\ & + condition \\ & + weekend \\ & + (condition : weekend) \\ & + (1|subject) \end{aligned} \tag{1}$$

Where *property_measure* refers to Period Mean, Period Std, Acrophase Mean, etc, *total_duration_d* refers to the total duration in days of all mid-week or circa-weekend segments for an individual, *condition* refers to whether the individual is a control or PWE, *weekend* refers to whether the *property_measure* is calculated over the mid-week (False) or circa-weekend (True) segments, and (*condition* : *weekend*) refers to the interaction between the *condition* and *weekend* terms. A random intercept was applied for each participant to account for systematic differences between participants that may arise due to chronotype or routines (work, exercise, etc). The outputs of this model are shown in Table S6.1 with p-values < 0.05 highlighted.

Additionally, to determine the relative importance of modelling the weekend effect, we performed likelihood ratio tests between the 'full' model (shown in Equation 1) and a 'reduced' model, that incorporates the weekend effect but not its interaction with condition (Equation 2) and a 'null' model that does not consider the weekend effect at all (Equation 3). p-values from these tests are reported in Table S6.2, with p-values < 0.05 highlighted.

$$\begin{aligned} property\_measure \sim & total\_duration\_d \\ & + condition \\ & + weekend \\ & + (1|subject) \end{aligned} \tag{2}$$

$$
\begin{aligned}
property\_measure \sim & total\_duration\_d \\
& + condition \\
& + (1|subject)
\end{aligned} \tag{3}
$$

In Figure S6.1, most intra-individual average and variability measures differ between weekdays and weekends in PWE and controls. We avoid direct comparison of effect sizes or p-values here, as there are substantially different sample sizes underlying the PWE *vs.* controls cohorts. Instead we report them in Figure S6.1 "as is".

In Figure S6.2, for each variability measure, there is a substantial difference in intra-individual variability of period and acrophase (Cohen's d ≥ 0.6), regardless of weekend or weekday. This confirms that the weekend effects observed in Fig S6.1 are not driving the results observed in Fig 3.

The MELR models outputs are reported in Table S6.1. Using the models to evaluate the interaction between the weekend and condition effects, there is weak evidence that intra-individual averages in Period and Amplitude are reduced on weekends only for PWE, although the associated p-values would not survive a correction for multiple comparison. No evidence of an interaction effect was seen in any of the intra-individual variability measures.

In Table S6.2, only Period and Amplitude Means have p-values below 0.05 in the reduced column, suggesting modelling the interaction between weekend and condition only can improve description of the data for these variables, which aligns with their entries in Table S6.1. However incorporation of at least the weekend effect alone is clearly necessary, as all p-values are $<< 0.05$ when comparing the null model (no incorporation of weekend effects) to the full.

Intra-individual circadian average and variability values appear to differ between weekdays and weekends for PWE and controls; but importantly, these differences do not drive the increased circadian variability we report in the main text. The weekday vs. weekend effect appear independently for both controls and PWE.

**Period Mean**	Coef.	Std.Err.	z	$P > \|z\|$	[0.025	0.975]
Intercept	24.005	0.042	570.531	**0.000**	23.922	24.087
C(Condition)[T.PWE]	0.073	0.045	1.607	0.108	-0.016	0.162
C(Weekend)[T.True]	0.013	0.055	0.243	0.808	-0.095	0.122
C(Condition)[T.PWE]:C(Weekend)[T.True]	-0.122	0.060	-2.027	**0.043**	-0.240	-0.004
total_duration_d	-0.000	0.000	-0.019	0.985	-0.000	0.000
Subject Var	0.004					

**Period Std**	Coef.	Std.Err.	z	$P > \|z\|$	[0.025	0.975]
Intercept	0.860	0.072	11.994	**0.000**	0.719	1.000
C(Condition)[T.PWE]	0.240	0.077	3.119	**0.002**	0.089	0.391
C(Weekend)[T.True]	0.105	0.062	1.712	0.087	-0.015	0.226
C(Condition)[T.PWE]:C(Weekend)[T.True]	0.019	0.067	0.290	0.772	-0.112	0.151
total_duration_d	-0.000	0.000	-1.364	0.173	-0.000	0.000
Subject Var	0.072	0.073				

**Acrophase Mean**	Coef.	Std.Err.	z	$P > \|z\|$	[0.025	0.975]
Intercept	15.312	0.470	32.555	**0.000**	14.390	16.233
C(Condition)[T.PWE]	0.137	0.504	0.273	0.785	-0.850	1.125
C(Weekend)[T.True]	0.265	0.113	2.349	**0.019**	0.044	0.487
C(Condition)[T.PWE]:C(Weekend)[T.True]	0.076	0.123	0.621	0.535	-0.165	0.318
total_duration_d	-0.001	0.001	-0.635	0.525	-0.002	0.001
Subject Var	4.884	2.063				

**Acrophase Std**	Coef.	Std.Err.	z	$P > \|z\|$	[0.025	0.975]
Intercept	0.550	0.052	10.651	**0.000**	0.449	0.651
C(Condition)[T.PWE]	0.178	0.055	3.204	**0.001**	0.069	0.286
C(Weekend)[T.True]	0.072	0.046	1.555	0.120	-0.019	0.162
C(Condition)[T.PWE]:C(Weekend)[T.True]	-0.025	0.050	-0.494	0.621	-0.123	0.074
total_duration_d	-0.000	0.000	-1.645	0.100	-0.000	0.000
Subject Var	0.036	0.049				

**Amplitude Mean**	Coef.	Std.Err.	z	$P > \|z\|$	[0.025	0.975]
Intercept	11.153	0.683	16.342	**0.000**	9.816	12.491
C(Condition)[T.PWE]	-0.549	0.731	-0.751	0.453	-1.981	0.884
C(Weekend)[T.True]	0.411	0.236	1.742	0.082	-0.051	0.873
C(Condition)[T.PWE]:C(Weekend)[T.True]	-0.543	0.257	-2.114	**0.035**	-1.047	-0.040
total_duration_d	-0.001	0.001	-0.460	0.645	-0.003	0.002
Subject Var	9.928	2.045				

**Amplitude Std**	Coef.	Std.Err.	z	$P > \|z\|$	[0.025	0.975]
Intercept	1.459	0.099	14.730	**0.000**	1.265	1.653
C(Condition)[T.PWE]	0.079	0.106	0.748	0.455	-0.129	0.288
C(Weekend)[T.True]	0.222	0.080	2.790	**0.005**	0.066	0.379
C(Condition)[T.PWE]:C(Weekend)[T.True]	-0.085	0.087	-0.982	0.326	-0.256	0.085
total_duration_d	-0.000	0.000	-0.814	0.415	-0.001	0.000
Subject Var	0.148	0.110				

Table S6.1: Summary of the mixed effects models produced by Equation 1.

	reduced	null
Period Mean	**0.032**	**0.0**
Period Std	0.772	**0.0**
Acrophase Mean	0.535	**0.0**
Acrophase Std	0.621	**0.021**
Amplitude Mean	**0.036**	0.098
Amplitude Std	0.327	**0.0**

Table S6.2: p-values for likelihood ratio tests comparing reduced (Equation 2) and null (Equation 3) models to the full (Equation 1) model for each property measure. $p < 0.05$ reject the null hypothesis and suggests the full model fits the data significantly better and should be used.

S6.2 Time of year effects

Seizures have been shown to exhibit seasonal variation, possibly clustering in winter (48), and seizure occurrences have not only been found to be phase-locked to circadian rhythms of heart rate for some PWE, but longer-term (weekly and monthly) rhythms of heart rate also (14). Here, we test whether intra-individual variability is associated with long-term seasonal trends, and whether this varies between PWE and controls.

To explore changes in intra-individual circadian average and variability over the year, 7-day segments (Figure 2G) were associated with the 'time of the year' (*toy*) at which they occurred. This is defined as $toy = \frac{day\_of\_year}{days\_in\_year}$ where day_of_year is an integer 1-365 (e.g. 1=1st January, 365=31st December) corresponding to the 4th (middle) circadian cycle of each 7-day segment, and days_in_year is 366 on leap years or 365 on regular years. Rather than producing a summary statistic for each participant by calculating the mean of circadian average and variability values across all segments as before, we can investigate whether these individual segment values vary depending on the time of year of the segment. This allows for a higher resolution in time along the year, and this is visualised for an example participant in Figure S6.3.

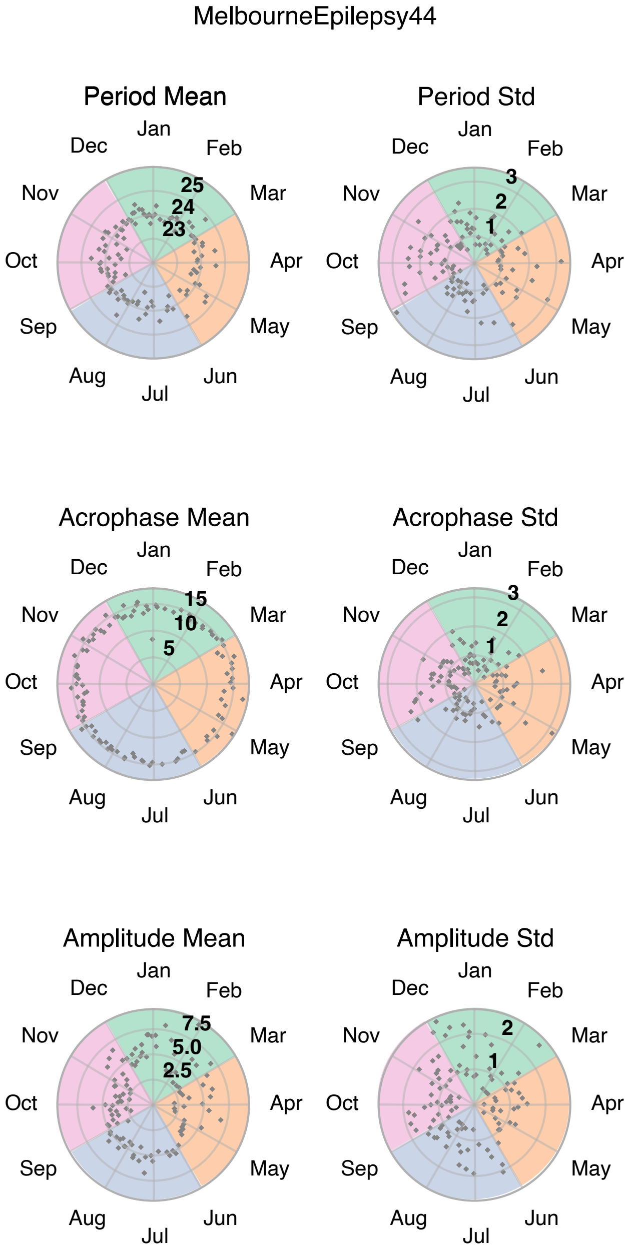

Figure S6.3: A visualisation of the data used in the MELR for one example participant. Each point corresponds to the circadian average or variability value for one 7-day segment, placed corresponding to its position within the year (data may span multiple years). Acrophase means have been corrected for local timezone and daylight saving time shifts. Coloured regions correspond to the seasons (Australian): Spring (Sept-Nov, pink), Summer (Dec-Feb, green), Autumn (Mar-May, orange), and Winter (Jun-Aug, blue).

To model the association between intra-individual average and variability of circadian properties in epilepsy and annual variations and determine the relative importance of these factors, a mixed-effects linear regression model (MELR) was used:

$$\begin{aligned} property\_measure \sim & condition \\ & + sin(toy\_radians) \\ & + cos(toy\_radians) \\ & + (condition : (sin(toy\_radians) + cos(toy\_radians))) \\ & +(1|subject) \end{aligned} \tag{4}$$

Where *property_measure* refers to Period Mean, Period Std, Acrophase Mean, etc, of a given segment, and *condition* refers to whether the individual is a control or PWE. As the time of year is a circular variable, it cannot be used directly in a linear regression model. To account for this, *cos* and *sin* of the month variable (after conversion to radians ($toy\_radians = toy \cdot 2\pi$)) were included. A significant association with either one or both of these terms can be interpreted as a seasonal effect being present. The interaction between these terms and condition was added via $condition : (sin(toy\_radians) + cos(toy\_radians))$. A random intercept was applied for each participant to account for systematic differences between participants that may arise due to chronotype or routines (work, exercise, etc); but here, also to account for different number of 7-day segments between subjects. The outputs of this model are shown in Table S6.3 with p-values < 0.05 highlighted.

Additionally, to determine the relative importance of modelling the time of year effect, we performed likelihood ratio tests between the 'full' model (shown in Equation 4) and a 'reduced' model, that incorporates the time of year effect but not its interaction with condition (Equation 5) and a 'null' model that does not consider the time of year effect at all (Equation 6). p-values from these tests are reported in Table S6.4 with p-values < 0.05 highlighted.

$$\begin{aligned} property\_measure \sim & condition \\ & + sin(toy\_radians) \\ & + cos(toy\_radians) \\ & + (1|subject) \end{aligned} \tag{5}$$

$$\begin{aligned} property\_measure \sim & condition \\ & + (1|subject) \end{aligned} \tag{6}$$

For the individual in Figure S6.3, period and acrophase averages are consistent over the course of the year, but other properties display seasonal variation.

Looking at the MELR output (Table S6.3), some seasonal effects were observed for intra-individual average and variability in PWE and controls, most pronounced in intra-individual average amplitude. However, we found no evidence that the reported increase in period and acrophase variability in PWE were driven by seasonal variations.

In Table S6.4, all rows bar average period have a p-value below 0.05 when compared to the null model, indicating that consideration of seasonal effects helps explain some variance in our circadian properties. Only average acrophase and amplitude had p-values below 0.05 in the reduced column, implying there may be some interaction between epilepsy and annual variation in intra-individual average acrophase and amplitude.

Intra-individual circadian average and variability values appear to vary subtly over the year in both PWE and controls. However, the relevance of this to epilepsy is not as clear: LR testing identified that average acrophase and amplitude are possibly altered in epilepsy, which could imply that PWE experience more of a seasonal shift in these properties than controls, but this needs to be explored more in future studies with substantially more controls. Most important to this work, we found no evidence of seasonal effects in intra-individual variability of period and acrophase. Thus our main results are unlikely to be driven by seasonal effects.

**Period Mean**	Coef.	Std.Err.	z	$P > \|z\|$	[0.025	0.975]
Intercept	24.038	0.024	989.150	**0.000**	23.990	24.085
C(Condition)[T.PWE]	0.016	0.026	0.611	0.541	-0.036	0.068
sin_toy_radians	0.027	0.018	1.518	0.129	-0.008	0.061
C(Condition)[T.PWE]:sin_toy_radians	-0.038	0.019	-2.022	**0.043**	-0.074	-0.001
cos_toy_radians	-0.006	0.019	-0.341	0.733	-0.043	0.031
C(Condition)[T.PWE]:cos_toy_radians	0.014	0.020	0.715	0.475	-0.025	0.053
Subject Var	0.009	0.004				

**Period Std**	Coef.	Std.Err.	z	$P > \|z\|$	[0.025	0.975]
Intercept	1.011	0.072	14.079	**0.000**	0.871	1.152
C(Condition)[T.PWE]	0.265	0.079	3.368	**0.001**	0.111	0.419
sin_toy_radians	-0.036	0.024	-1.531	0.126	-0.083	0.010
C(Condition)[T.PWE]:sin_toy_radians	0.024	0.025	0.966	0.334	-0.025	0.073
cos_toy_radians	-0.005	0.025	-0.210	0.833	-0.055	0.045
C(Condition)[T.PWE]:cos_toy_radians	-0.014	0.027	-0.537	0.591	-0.067	0.038
Subject Var	0.127	0.030				

**Acrophase Mean**	Coef.	Std.Err.	z	$P > \|z\|$	[0.025	0.975]
Intercept	15.394	0.405	37.978	**0.000**	14.599	16.188
C(Condition)[T.PWE]	0.031	0.446	0.069	0.945	-0.843	0.905
sin_toy_radians	0.076	0.066	1.149	0.250	-0.053	0.205
C(Condition)[T.PWE]:sin_toy_radians	-0.104	0.069	-1.504	0.132	-0.240	0.032
cos_toy_radians	0.084	0.070	1.194	0.232	-0.054	0.222
C(Condition)[T.PWE]:cos_toy_radians	0.231	0.074	3.141	**0.002**	0.087	0.376
Subject Var	4.750	0.391				

**Acrophase Std**	Coef.	Std.Err.	z	$P > \|z\|$	[0.025	0.975]
Intercept	0.867	0.082	10.616	**0.000**	0.707	1.027
C(Condition)[T.PWE]	0.252	0.090	2.815	**0.005**	0.077	0.427
sin_toy_radians	-0.017	0.026	-0.662	0.508	-0.069	0.034
C(Condition)[T.PWE]:sin_toy_radians	-0.012	0.028	-0.436	0.663	-0.066	0.042
cos_toy_radians	-0.009	0.028	-0.313	0.754	-0.064	0.046
C(Condition)[T.PWE]:cos_toy_radians	-0.026	0.029	-0.873	0.383	-0.084	0.032
Subject Var	0.166	0.035				

**Amplitude Mean**	Coef.	Std.Err.	z	$P > \|z\|$	[0.025	0.975]
Intercept	10.951	0.592	18.511	**0.000**	9.791	12.110
C(Condition)[T.PWE]	-0.692	0.651	-1.062	0.288	-1.968	0.585
sin_toy_radians	0.289	0.096	3.010	**0.003**	0.101	0.477
C(Condition)[T.PWE]:sin_toy_radians	-0.024	0.101	-0.239	0.811	-0.222	0.174
cos_toy_radians	0.678	0.103	6.603	**0.000**	0.476	0.879
C(Condition)[T.PWE]:cos_toy_radians	-0.335	0.107	-3.122	**0.002**	-0.546	-0.125
Subject Var	10.119	0.541				

**Amplitude Std**	Coef.	Std.Err.	z	$P > \|z\|$	[0.025	0.975]
Intercept	2.013	0.114	17.689	**0.000**	1.790	2.236
C(Condition)[T.PWE]	-0.027	0.125	-0.216	0.829	-0.271	0.217
sin_toy_radians	0.010	0.039	0.268	0.788	-0.066	0.086
C(Condition)[T.PWE]:sin_toy_radians	0.011	0.041	0.266	0.790	-0.069	0.091
cos_toy_radians	0.100	0.042	2.416	**0.016**	0.019	0.182
C(Condition)[T.PWE]:cos_toy_radians	-0.049	0.043	-1.136	0.256	-0.135	0.036
Subject Var	0.316	0.046				

Table S6.3: Summary of the mixed effects models produced by Equation 4.

	reduced	null
Period Mean	0.091	0.106
Period Std	0.526	**0.028**
Acrophase Mean	**0.002**	**0.0**
Acrophase Std	0.634	**0.0**
Amplitude Mean	**0.008**	**0.0**
Amplitude Std	0.497	**0.0**

Table S6.4: p-values for likelihood ratio tests comparing reduced (Equation 5) and null (Equation 6) models to the full (Equation 4) model for each property measure. $p < 0.05$ reject the null hypothesis and suggests the full model fits the data significantly better and should be used.
